# JAK/STAT信号通路在肺癌中的研究进展

**DOI:** 10.3779/j.issn.1009-3419.2019.01.09

**Published:** 2019-01-20

**Authors:** 昕 杨, 哲 唐, 鹏 张, 莉 张

**Affiliations:** 1 430030 武汉，华中科技大学同济医学院附属同济医院肿瘤科 Department of Oncology, Tongji Hospital, Tongji Medical College, Huazhong University of Science and Technology, Wuhan 430030, China; 2 430030 武汉，华中科技大学同济医学院附属同济医院泌尿外科 Department of Urology, Tongji Hospital, Tongji Medical College, Huazhong University of Science and Technology, Wuhan 430030, China

**Keywords:** JAK-STAT信号通路, 肺肿瘤, 耐药, 抑制剂, JAK/STAT signaling pathway, Lung neoplasms, Drug resistance, Inhibitors

## Abstract

Janus激酶（Janus kinase, JAK）/信号转导子和转录活化子（signal transducer and activator of transcription, STAT）信号通路是细胞因子信号传导的下游通路，调控细胞的发育、分化、增殖、凋亡等，不仅参与调节正常的生理过程，在肿瘤的发生发展中也起着重要作用，尤其是在血液系统肿瘤中意义重大。近年来，随着对JAK/STAT信号通路研究的深入，人们发现该通路在实体肿瘤的发生发展中也扮演关键角色。本文就近年来JAK/STAT信号通路参与肺癌发生发展、肺癌转移、肺癌耐药机制形成以及靶向该通路的抑制剂在肺癌治疗中的应用现状进行综述。

## Abstract (en)

Janus kinase (JAK)/signal transducer and activator of transcription (STAT) signaling pathway is one of the downstream pathways of cytokine signaling transduction. It regulates cell development, differentiation, proliferation, apoptosis and so on. The pathway is not only involved in the regulation of normal physiological processes, but also significant in the development of tumors, especially in hematologic malignancies. In recent years, with the further research of JAK/STAT signaling pathway, it has been found that the pathway also plays a key role in the development of solid tumors. Here we reviewed the research advances of JAK/STAT signaling pathway in lung cancer, especially the mechanisms of development, metastasis and drug resistance, and the application of inhibitors which targeting JAK/STAT signaling pathway in the treatment of lung cancer.

Janus激酶（Janus kinase, JAK）/信号转导子和转录活化子（signal transducer and activator of transcription, STAT）信号通路最开始是作为细胞因子受体的下游转导途径被发现。该信号通路在正常的生理过程中有重要作用，主要调控血液细胞对细胞因子的反应，同时也是一些激素受体，如生长激素和催乳素受体的下游途径，参与了细胞的发育、分化、增殖、凋亡及免疫调节等重要生物学过程。

早先人们发现在骨髓增生性疾病及白血病中存在*JAK*基因的持续激活，而抑制*JAK*基因则可以使肿瘤消退，从而证实了*JAK*基因在肿瘤进展中的作用。近年来，随着对JAK/STAT信号通路的深入研究，人们发现该信号通路不仅参与血液系统肿瘤的发生发展，在实体肿瘤如肺癌中也有重要作用，本文就其在肺癌中的研究进展作一综述。

## 1 JAK/STAT信号通路的组成

JAK/STAT信号通路主要由三个成分组成，即酪氨酸激酶相关受体、JAK和STAT。酪氨酸激酶相关受体本身不具有激酶活性，但胞内段具有JAK的结合位点，当受体与配体（包括多种细胞因子和生长因子，如白介素、集落刺激因子、表皮生长因子、血小板衍生因子、干扰素等）结合后，JAK活化，催化结合在受体上的STAT蛋白发生磷酸化修饰，活化的STAT蛋白以二聚体的形式进入细胞核内与靶基因结合，调控基因的转录^[1]^。

Janus激酶的名字来源于其包含两个激酶样结构域的结构（Janus是罗马神话中的一个双面神）。迄今为止，JAK家族共发现有4个家族成员即JAK1、JAK2、JAK3和酪氨酸激酶2（tyrosine kinase 2, TYK2）。不同的家族成员选择性地结合在不同的受体上，从而发挥不同的生理学作用，这种选择性的作用方式使得JAK抑制剂可以相对特异性地应用于疾病治疗。

STAT家族一共包括STAT1、STAT2、STAT3、STAT4、STAT5a、STAT5b和STAT6等7个家族成员。活化的JAKs能激活特定的STAT家族成员，但也可以与其他的STAT家族成员相互作用，形成复杂的调控网络。STAT家族成员磷酸化后进入细胞核，从而调控基因的转录。正常情况下，STATs可以被快速而短暂地激活，活化的STATs进入细胞核后作用数分钟至几小时后，会被脱磷酸化而使STATs失活，STATs重新被转运回胞浆等待再次被激活（图 1）。

**1:**
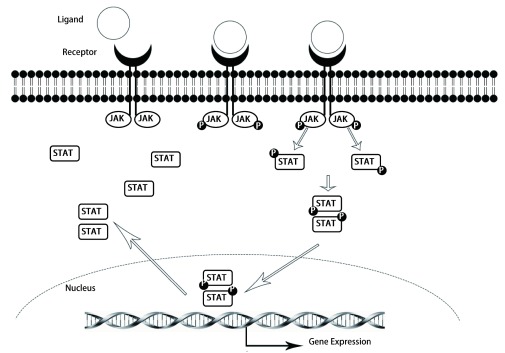
JAK/STAT信号通路 The signaling pathways of JAK/STAT

对于STAT家族成员调控基因转录的机制，目前认为，STAT家族成员可以结合上万个基因组的位点，调节数以千计的编码蛋白基因的表达，还通过影响非编码RNA的表达，间接调控基因转录。JAK家族与STAT家族的多个成员共同构成了多条信号通路，如JAK1/STAT6、JAK2/STAT3和JAK2/STAT5等。

## 2 JAK/STAT信号通路与肺癌

肺癌是全球癌症死亡的主要原因，也是中国发病率和死亡率最高的恶性肿瘤，并且发病率有逐年上升的趋势。由于早期诊断不足，大部分患者在初诊时已处于中晚期，无法行手术治疗或根治性放疗，预后较差^[2]^。近年来，随着人们对肺癌的深入研究，新化疗药物、靶向治疗、免疫治疗等治疗方法的出现，肺癌的生存率有所提高。早期诊断和规范治疗能使患者最大获益，同时仍需进一步加深肺癌的基础研究。

Leonard等^[3]^最先提出JAK/STAT信号通路家族成员的组成型激活与肿瘤的发生发展有关。JAK/STAT信号通路可以被多种机制所激活，如自分泌或旁分泌产生的细胞因子与受体作用，JAK家族成员或上游原癌基因的突变导致JAKs的激活，继而激活STAT家族成员。极少数情况下，STAT的基因突变也会导致其自身的激活。JAK/STAT信号通路的持续激活与肺癌的发生发展、转移以及耐药的发生有密切联系。

### 2.1 JAK/STAT信号通路与肺癌的发生发展

JAK/STAT1、JAK/STAT3、JAK/STAT5是在肿瘤发生发展中发挥作用的三条经典通路。JAK家族成员在肺癌组织中的高表达以及基因突变与肿瘤的发生发展有关。Xu等^[4]^发现*JAK2*基因在癌组织中表达上调，并且与淋巴结转移相关。上调JAK2的表达会增强肿瘤细胞增殖、转移和侵袭的能力，下调JAK2的表达则作用相反。进一步研究还发现肺腺癌组织中存在*JAK2*基因的突变，推测JAK2的突变也与肺癌进展、预后较差以及耐药有关。另一项研究结果^[5]^显示肺癌中*JAK2*、*JAK3*突变与PD-L1的表达高低有关，携带*JAK3*基因突变的患者可能从免疫治疗获益。Liu等^[6]^在非小细胞肺癌（non-small cell lung cancer, NSCLC）患者中发现JAK1的磷酸化水平显著增高，并且其高表达与不良预后相关，可以作为不良预后的预测因子。

虽然JAK家族成员在肺癌的发生发展中有一定意义，但主要通过影响STATs的活性发挥作用。多项研究^[7-10]^分析了NSCLC中各信号通路中STAT家族成员的表达量变化，结果显示，与正常组织相比，癌组织中STAT1、STAT3、STAT5的表达量有显著性改变，其中STAT1的表达显著降低，而STAT3与STAT5的表达量显著升高（STATs家族在NSCLC组织中的表达量分析及其潜在靶基因的研究）。除此之外，Pastuszak等^[11]^在NSCLC中还检测到磷酸化STAT6的高表达。

#### 2.1.1 JAK/STAT1信号通路与肺癌

STAT1与干扰素（interferon, IFN）信号通路密切相关，发挥抑制细胞生长以及促进细胞凋亡的作用。*STAT1*基因敲除小鼠对自发或诱导的肿瘤较正常小鼠敏感，提示STAT1可能有抑癌作用。Kachroo等^[7]^应用NSCLC细胞系研究发现，白细胞介素-27（interleukin-27, IL-27）通过STAT1诱导上皮细胞转化和抑制促血管生成因子的生成。赵嘉璐等^[12]^发现过表达STAT1可抑制NSCLC H1299细胞增殖，马源等^[13]^发现*STAT1*基因转染可以抑制人肺腺癌裸鼠移植瘤的生长。这些研究均显示，STAT1可以发挥抑制肺癌发生发展的作用。

#### 2.1.2 JAK/STAT3信号通路与肺癌

JAK/STAT3是目前被研究最多的信号通路，STAT3的持续激活与细胞恶性转化密切相关，参与多种肿瘤的发生发展，是目前国内外对STAT家族成员在肺癌研究中的主要靶点。Yu等^[8]^研究发现，在肺癌中可以检测到STAT3的组成型激活，因此认为其与肺癌的发生发展密切相关。后续研究进一步发现大约55%的NSCLC患者以及大多数NSCLC细胞系中，均存在STAT3的异常活化。这种活化更常见于小肿瘤患者、较短吸烟史患者以及肺腺癌患者。Wu等^[14]^通过收集、分析既往63项相关研究，发现STAT3表达水平的升高与肺癌不良预后有关，表现在高表达STAT3的患者肿瘤分期更晚，3年总体生存率更低。Xu和Tong等^[15, 16]^的研究分析也得出了类似的结论。Pfeiffer等^[47]^在针对小细胞肺癌的研究中发现，所有的小细胞肺癌患者（10/10）以及绝大部分小细胞肺癌细胞系（6/7）中，均检测出磷酸化STAT3的高表达，但目前没有更大型的研究进一步证实STAT3的激活与小细胞肺癌的关系。虽然绝大部分研究结果均表明STAT3的过度激活促进肿瘤的发生发展，但是Grabner等^[17]^研究发现下调STAT3的表达反而促进*KRAS*突变的肺腺癌的形成。不过值得注意的是，该研究中应用shRNA导致STAT3的功能近乎完全缺失，才观察到促进肿瘤发展的现象，一般的治疗方法下调肿瘤中STAT3的活性是达不到这种效果的，其机制尚有待研究。

#### 2.1.3 JAK/STAT5信号通路与肺癌

STAT5在肺癌组织的表达量也明显升高。Sánchez-Ceja等^[9]^发现磷酸化STAT5在多种类型肺癌中均存在不同程度的高表达，并且其表达水平可能与肺癌的分期有关。Pastuszak等^[11]^的研究也表明在NSCLC患者，尤其是分期为pT2的患者中，磷酸化STAT5呈现明显高表达。

### 2.2 JAK/STAT信号通路与肺癌转移

研究发现，JAK/STAT信号通路与血液系统肿瘤以及包括肺癌在内的多种实体肿瘤的远处转移密切相关。Jiang等^[18]^应用免疫组织化学的方法分析癌组织样本中STAT3活性与癌组织临床病理特点的关系，发现磷酸化STAT3的免疫反应性与性别、吸烟史、表皮生长因子受体（epidermal growth factor receptor, *EGFR*）突变类型、肿瘤临床分级和淋巴结转移均相关，提示STAT3的活性在肺癌淋巴结转移中可能有一定作用。Sun等^[19]^也在NSCLC中发现JAK1/STAT1活性与肺癌分期和淋巴结转移呈现正相关。

许多研究者都关注JAK/STAT的活性影响肺癌转移的机制。Chuang等^[20]^通过携带人类转移肺腺癌组织的基因工程小鼠模型，应用腺病毒示踪肿瘤组织来源，鉴定出CD109在肺腺癌转移过程中发挥重要作用，敲减该分子可以显著降低肿瘤转移。同时他们还证明，CD109依赖JAK/STAT3信号通路发挥作用，敲减STAT3或应用JAK抑制剂可以抑制肿瘤转移，提示JAK/STAT3信号通路在肺癌转移中发挥重要作用。Lin等^[21]^在体外研究STAT3促进人肺癌细胞侵袭性的机制时发现，STAT3通过上调miR-92a的活性，从而靶向并下调RECK的表达，使肿瘤细胞据有更强的侵袭性。

虽然目前JAK/STAT信号通路参与肺癌转移的机制尚未完全阐明，但很多研究显示降低JAK/STAT活性可以抑制肺癌转移，提示JAK/STAT可能是一个抑制肺癌转移的治疗靶点。

## 3 JAK/STAT信号通路与肺癌靶向治疗耐药

靶向治疗的出现给肺癌患者带来了希望，可以延长患者的无疾病进展期（progression-free survival, PFS）及总体生存率（overall survival, OS），改善患者的生活质量。以最常见的NSCLC为例，大约2/3的患者均携带驱动基因突变，如*EGFR*、*ALK*、*ROS1*、*BRAF*等突变的基因，是靶向药物治疗的良好靶点^[22]^。但经过一段时间的治疗，药物的作用会减弱甚至无效，出现耐药。

耐药可以分为三种类型：固有耐药、适应性耐药和获得性耐药^[23]^。JAK/STAT信号通路主要参与了适应性耐药和获得性耐药的机制形成。适应性耐药指的是肿瘤对靶向治疗有反应，但是部分肿瘤细胞会在药物治疗早期发生适应性变化以存活；获得性耐药可能来源于最初肿瘤细胞中已经存在的基因突变的选择性联合，也可来源于在靶向药物的作用下获得了新的基因突变。

JAK/STAT信号通路的激活可以作为一种适应性应答，发生在EGFR-TKI治疗*EGFR*基因突变的NSCLC的早期。Yao等^[24]^发现IL-6激活的gp130/JAK/STAT3信号通路降低了NSCLC H1650细胞对TKI药物的敏感性。Harada等^[48]^在研究中发现，对厄洛替尼耐药的肺癌细胞存在磷酸化JAK2的高表达，而Kim等^[25]^也在对阿法替尼耐药的T970M突变的NSCLC细胞中检测到STAT3的高表达，推测JAK2、STAT3的激活可能参与获得性耐药的机制形成。

一些基础研究的结果显示，联合应用JAK或STAT3抑制剂和EGFR-TKI可以产生更显著的治疗作用。肿瘤细胞自分泌的白细胞介素6（interleukin 6, IL-6）作用于自身的受体可以增强JAK/STAT3的活性，在小鼠肿瘤模型中应用IL-6受体的中和抗体可以抑制肿瘤生长^[25]^。Gao等^[26]^在体外实验中发现，高表达的磷酸化STAT3在TKI耐药的NSCLC中持续存在，说明磷酸化STAT3在介导肺癌TKI耐药上有重要作用；单独应用JAK抑制剂可以减小肿瘤异种移植物的形成，联合应用JAK抑制剂和EGFR-TKI则能够使已经耐药的肺腺癌细胞再次对EGFR-TKI敏感。Chiu等^[27]^也发现抑制STAT3的活性可以增强吉非替尼耐药的NSCLC细胞对药物的敏感性。Blakely和Lee等^[28, 29]^在EGFR-TKI治疗肿瘤的早期就观察到了JAK/STAT3信号通路的适应性激活，因此可能有必要提前应用JAK和/或STAT抑制剂联合EGFR-TKI进行治疗。在Yu等^[30]^报道的一项临床试验中，对既往使用厄洛替尼治疗后出现进展的22例患者，联合应用JAK抑制剂鲁索替尼和厄洛替尼进行治疗，观察到1例携带*EGFR* T790M突变的患者出现了部分缓解。联合用药对于T970M突变介导的TKI耐药患者可能是一项有意义的尝试。目前，有一项临床试验（NCT02917993）将JAK抑制剂Itacitinib（INCB-39110）联合奥希替尼应用于*EGFR*-T790M突变的肺癌患者，有效性仍有待检验。

## 4 JAK/STAT信号通路抑制剂在肺癌中的应用

随着对JAK/STAT信号通路在肺癌中作用理解的深入，一些JAK/STAT信号通路抑制剂也逐渐应用于基础研究和临床实验，以期达到治疗肺癌的效果。JAK抑制剂按照其作用机制可以分为3种类型^[31]^：Ⅰ型抑制剂作用于JAKs激酶结构域活性构象的ATP结合位点，Ⅱ型抑制剂作用于激酶结构域非活性构象中结合ATP的袋装结构，Ⅲ型为变构抑制剂，作用于活性构象以外的结合位点，理论上来说对突变型JAKs更有效果。目前进入临床研究的JAK抑制剂均为Ⅰ型抑制剂。针对STATs的抑制剂目前研究相对有限。

### 4.1 鲁索替尼（Ruxolitinib）

鲁索替尼是美国FDA批准上市的首个JAK抑制剂，可以选择性地抑制JAK家族成员——JAK1和JAK2，其作用机制是竞争性结合JAK1/2激酶结构域催化亚基上的ATP结合位点，从而抑制JAK1/2的活性。鲁索替尼被美国FDA批准用于骨髓纤维化和真性红细胞增多症，但很多研究表明鲁索替尼用于实体肿瘤治疗也有一定效果。

Tavallai等^[32]^在体外实验中发现联合应用鲁索替尼和ERBB抑制剂（如阿法替尼）可以杀伤如肺癌、乳腺癌、卵巢癌等肿瘤细胞，其机制可能是通过使AKT、mTORC1、mTORC1、STAT3和STAT5失活，继而下调MCL-1、BCL-XL等抗凋亡蛋白的活性，从而增强药物杀伤肿瘤细胞的作用。Hu等^[33]^通过细胞实验和动物实验表明JAK/STAT信号通路与肺癌顺铂耐药密切相关，鲁索替尼可以克服NSCLC对顺铂的耐药性，联合应用鲁索替尼可以增强肺癌对铂类药物的敏感性。Yu等^[30]^首次报道了一项联合应用鲁索替尼和厄洛替尼的临床试验，试验共招募22例有*EGFR*突变并对EGFR-TKI耐药的肺癌患者，检测联合用药的安全性和有效性。结果观察到1例携带*EGFR* T790M突变的患者持续12个月的部分缓解，总体中位无进展生存时间（PFS）为2.2个月（95%CI: 1.4-4.1），患者对于药物的耐受性良好。另一项临床试验（NCT02145637）联合应用鲁索替尼和阿法替尼治疗肺癌，结果观察到40%的部分反应率（partial response, PR），86.7%的疾病控制率（disease control rate, DCR），总体中位PFS为8.8个月（95%CI: 1.8-15.8）。

### 4.2 AZD1480

AZD1480是一个主要抑制JAK1/2活性的小分子化合物，同时对Trk-A、Aurora-A、Flt4以及FGFR1等激酶也有一定的抑制作用。Hedvat等^[34]^首先阐明AZD1480通过抑制JAK2/STAT3信号通路，在小鼠荷瘤模型上发挥抑制人类实体肿瘤生长的作用，实验中选择的实体肿瘤细胞系均检测到STAT3的高表达。Xin等^[35]^通过在小鼠尾静脉注射Renca细胞或在小鼠侧腹部植入含人肺腺癌细胞系Calu-6的基质胶，进一步研究AZD1480抑制肺癌血管再生及转移的作用。结果表明，AZD1480可以显著抑制小鼠肺中Renca肿瘤血管再生以及抑制肺癌的淋巴结转移，同时肿瘤组织中p-STAT3、VEGF和MMP9的活性明显下降。还有两项分别针对小细胞肺癌^[36]^和*EGFR*突变导致的肺癌^[37]^的研究也表明，AZD1480也可以抑制肿瘤的生长。Plimack等^[38]^报道了一项将AZD1480应用于治疗实体肿瘤的临床试验的初步结果，观察到一例肺癌患者服用AZD1480病情稳定时间超过4个月（145 d），但由于试验中出现神经系统副作用以及总体有效性的不足，这项试验没能继续。AZD1480治疗实体肿瘤的效果仍有待进一步研究。

### 4.3 AG490

AG490是一种人工合成的JAK2小分子抑制剂，可通过抑制JAK2磷酸化从而抑制STAT3的活化。多项研究显示，AG490可以显著阻断肿瘤中持续活化的JAK2/STAT3信号通路，从而发挥抑制肺癌细胞生长^[39]^及侵袭、抑制肿瘤组织血管再生^[40]^、抑制上皮间质转化^[41]^等作用。但是，目前的研究尚停留在细胞层面，AG490对肺癌的治疗作用仍需进一步探究。

### 4.4 STATs抑制剂

虽然目前有很多药物设计方法被应用于STAT家族成员尤其是STAT3抑制剂的设计，但大部分STAT3抑制剂仍处于基础研究阶段，在肺癌治疗上进展有限。Zhang等^[42]^运用计算机辅助技术发现了一种可以结合STAT3结构中Src同源区（SH2）的抑制剂——BP-1-102，并通过动物实验证明该抑制剂能抑制肺癌异种移植物的生长。香豆素类化合物（如狭叶芸香的提取物——Chalepin）是一类具有广泛生理作用的天然产物，对肿瘤有一定的抑制活性，研究证实该类化合物有抑制肺癌的作用，而抑制STAT3的活性是其发挥抑癌作用的机制之一^[43]^。Blaskovich等^[44]^通过高通量筛选发现Cucurbitacin I（JSI-124）是STAT3的抑制剂，也能抑制JAKs的活性，且选择性作用于JAK/STAT3信号通路，并初步证实该抑制剂可以抑制裸鼠体内肺癌组织的生长。Guo等进一步研究Cucurbitacin I的作用机制，结果显示Cucurbitacin I可以抑制STAT3活化，还能增强STAT1的表达^[45]^。但Cucurbitacin I并不是直接抑制JAK2/STAT3信号通路的激活，而是通过干扰肌动蛋白丝的功能，从而调节JAK/STAT信号通路的活性，发挥抑癌作用。Wong等^[46]^首次报道了一项STAT3小分子抑制剂——OPB-51602应用于实体肿瘤治疗临床试验的初步结果，试验一共招募了25例NSCLC患者，其中两例患者达到部分缓解，这2例患者均携带*EGFR*基因突变，且既往接受EGFR-TKI治疗。STATs抑制剂应用于肺癌治疗还需要更深入的研究。

## 5 结论

虽然目前人们已经了解JAK/STAT信号通路在肺癌发生、发展中的重要性，但对其作用机制尚不完全明确。对JAK/STAT信号通路的深入研究，有助于进一步加深对肺癌的认识，更加明确肺癌发生、发展、耐药的机制，为开发肺癌治疗的新方法提供理论依据，为临床上治疗肺癌提供新的思路。
